# Subadult ravens generally don't transfer valuable tokens to conspecifics when there is nothing to gain for themselves

**DOI:** 10.3389/fpsyg.2015.00885

**Published:** 2015-06-30

**Authors:** Jorg J. M. Massen, Megan Lambert, Martina Schiestl, Thomas Bugnyar

**Affiliations:** ^1^Department of Cognitive Biology, University of ViennaVienna, Austria; ^2^Department of Psychology, University of YorkYork, UK; ^3^Haidlhof Research Station, University of Vienna and University of Veterinary MedicineVienna, Austria

**Keywords:** prosociality, other-regard, ravens, altruism, cooperation

## Abstract

The extent to which humans help each other is extraordinary in itself, and difficult to explain from an evolutionary perspective. Therefore, there has been a recent surge in studies investigating the evolution of prosocial behavior using a comparative approach. Nevertheless, most of these studies have focused on primates only, and little is known about other animal orders. In a previous study, common ravens (*Corvus corax*) have been shown to be indifferent to the gains of conspecifics. However, this may have been due to the experimental set-up, as many studies that use different set-ups report conflicting results within the same species. We therefore tested ravens' prosocial tendencies in a different set-up; i.e., we tested whether sub-adult ravens would transfer a token to a partner and, thereby, provide the partner with the opportunity to exchange a token for a reward. To control and test for effects of partner identity, we tested eight individuals both in a dyadic and in a group setting. Our results show that in general the ravens in our experiment did not show other-regarding preferences. However, some acts of helping did occur spontaneously. We discuss what could be the causes for those sporadic instances, and why in general prosocial tendencies were found to be almost lacking among the ravens in this set-up.

## Introduction

Recently, socio-ecological studies of behavior have shifted their focus from “selfish” behaviors to those considered to be more altruistic (Silk and House, 2011). These altruistic behaviors are particularly interesting from an evolutionary perspective, as they are likely to increase the recipient's fitness at a cost to the actor (de Waal, 2008). The associated costs of altruisms can be overcome by kin-selection (Hamilton, 1964), or through reciprocity (Trivers, 1971). However, both explanations are at the ultimate level, and do not provide insight into the proximate motivations underlying this behavior (Tinbergen, 1963). Nevertheless, humans show a wide array of altruistic acts also toward non-kin (Fehr and Fischbacher, 2003), including prosocial behavior, which may be defined as “behavior that benefits others at no or low cost to the actor” (Silk and House, 2011). Consequently, there has been a surge in studies investigating the evolution of such behavior either through models (e.g., de Vos et al., 2001; Boyd et al., 2010; Forber and Smead, 2014) or by comparing our behavior with that of other primates.

The first experimental evidence for prosocial preferences in non-humans was documented in common marmosets (*Callithrix jacchus*: Burkart et al., 2007). Like humans, common marmosets are characterized by a cooperative breeding system in which several individuals will contribute to the care of offspring that are often not their own, or that are not even related to themselves (Burkart et al., 2009; Hrdy, 2009). As providing food to non-relatives obviously requires some sort of prosocial tendency, it was hypothesized that prosocial behavior had evolved as a consequence of this socio-ecological adaptation (i.e., the Cooperative Breeding Hypothesis, Burkart et al., 2007, 2009). Corroborating this hypothesis, a recent study on 15 primate species showed that the degree of allomaternal care per species predicts group provisioning by a single individual of that species (Burkart et al., 2014). Nevertheless, prosocial behavior has, albeit to a lesser degree than in humans and marmosets, also been experimentally shown in many other primate species (brown capuchin monkeys, *Cebus apella*: de Waal et al., 2008; long-tailed macaques, *Macaca fascicularis*: Massen et al., 2010; cotton-top tamarins, *Saguinus oedipus*: Cronin et al., 2010; bonobos, *Pan paniscus*: Hare and Kwetuenda, 2010; chimpanzees, *Pan troglodytes*: Horner et al., 2011; and orang-utans, *Pongo pygmaeus*: Liebal et al., 2014) including many that do not breed cooperatively. This suggests that all primates may share at least a basic tendency for pro-sociality through common ancestry, and little can be said about the evolutionary pressures that led to the evolution of this behavior in this common ancestor. Comparisons with non-primate species thus seem warranted (see also Thornton and McAuliffe, 2015).

Among birds, the corvid family shares several socio-ecological characteristics with primates (Emery, 2006) that seem to have led to similar cognitive capacities (Emery and Clayton, 2004). In a recent study, Schwab et al. (2012) showed that jackdaws, *Corvus monedula*, can behave prosocially in an experimental set-up, although only when the recipient shows particular interest in the reward. Intriguingly, however, the closely related common raven did not demonstrate such preferences in a similar set-up (Di Lascio et al., 2013). This deviation between two closely related species might highlight important socio-ecological differences that may help explain the evolution of prosociality. For example, jackdaws may breed in colonies (Johnsson, 1994), whereas ravens exclusively breed in territorial pairs (Heinrich, 1999). Although the jackdaws do not show allo-parental care, their openness to communal breeding does require more intraspecific tolerance than the territorial ravens, which may have led to the evolution of more pronounced prosocial tendencies (cf. Burkart et al., 2014). Alternatively, this deviation may result from methodology (Tan et al., 2015). For example, several studies on chimpanzees and one on bonobos failed to show prosociality in our closest living relatives when using a two-choice paradigm featuring visible food rewards (Silk et al., 2005; Jensen et al., 2006; Vonk et al., 2008; Amici et al., 2014; Tan et al., 2015), whereas others readily demonstrated prosociality in chimpanzees and bonobos using either a helping task (Warneken and Tomasello, 2006; Warneken et al., 2007; Yamamoto et al., 2009, 2012; Hare and Kwetuenda, 2010; Greenberg et al., 2011; Hamann et al., 2011; Melis et al., 2011; Tan and Hare, 2013), or a slightly adapted two-choice paradigm that uses tokens as a means to retrieve rewards through an exchange with an experimenter (Horner et al., 2011, but see Amici et al., 2014). Similarly, in human studies results are conflicting; for example, slight adaptations to the popular public-goods game have resulted in a breakdown of cooperative behavior, leading scientists to question the prosocial nature of humans in general (Kümmerli et al., 2010; Burton-Chellew and West, 2013). In addition, pro-sociality in children has been shown to be rather unstable at a young age (Li et al., 2013), and conflicting results have been found with different set-ups (Burkart and Rueth, 2013). Most of this inconsistency has been attributed to differences in the attentional and cognitive demands of the different tasks (Warneken and Tomasello, 2006; Horner et al., 2011; Burkart and Rueth, 2013; Amici et al., 2014; House et al., 2014).

Therefore, we re-examined prosocial preferences of common ravens using a different set-up than the prosocial choice task of Di Lascio et al. (2013). In particular, in the current study we tested eight subadult ravens in a helping task; i.e., we tested their willingness to offer a conspecific a token, which, in contrast to the actor itself, the recipient could exchange for a food reward with an experimenter (cf. Dufour et al., 2009; Pelé et al., 2009). We chose to use subadult subjects since subadult ravens in the wild live in complex social flocks, and form strong and stable social bonds (not only with future pair-mates) (Fraser and Bugnyar, 2010). Moreover, subadult ravens are known to show and offer each other objects in their everyday life (Pika and Bugnyar, 2011), making the setup we use possibly more intuitive for the ravens than the set-up of Di Lascio et al. (2013).

Our set-up allowed to investigate whether the behavior of the partner in the test, the identity of the tested individual and the composition of the dyad could predict whether an individual would offer a conspecific a token or not. The behavior of recipients has been shown to both positively as well as negatively influence the pro-social choices of subject; e.g., whereas direct requests or harassment of recipients decreased prosocial preferences of chimpanzees (Horner et al., 2011), when the recipient only showed interest this did increased prosocial preferences in the same study (Horner et al., 2011) and also in a study on jackdaws (Schwab et al., 2012). Similarly, the identity of the subject and the composition of the dyad could influence a subject's tendency to choose prosocially in an experiment. For example high ranking long-tailed macaques, *Macaca fascicularis*, are pro-social, whereas low ranking ones are a-social (Massen et al., 2010). However, the latter ones choose to be prosocial toward the highest ranking of two potential recipients (Massen et al., 2011). For a detailed overview of potential factors influencing prosociality and the results on different species so far, see Cronin (2012).

Our study consisted of two experimental settings: in Experiment 1, we paired each individual with a fixed partner, whereas in Experiment 2 the actor could potentially choose its own recipient, while the whole social group was present. Moreover, in Experiment 2 we tested whether, if the ravens would be prosocial in our tests, they would also forego a reward themselves to provide one to another, showing costly pro-sociality (cf. Sterck et al., 2015).

## Methods

### Subjects, housing, and ethics

Experiment 1 was conducted between February 2013 and July 2013, and Experiment 2 between April 2014 and June 2014. We tested eight subadult captive ravens consisting of 5 males and 3 females that were around 1 year old at the time when we conducted Experiment 1. All were housed in one social group containing 10 birds in total at the Haidlhof Research Station, Bad Vöslau, Austria. All ravens from this group were born in 2012 and originated from four captive breeding pairs in zoos (Zoo Wels, Austria; Tierpark Haag, Austria; Nationalpark Bayrischer Wald, Germany; Spanga, Stockholm, Sweden). All birds were hand-raised identically in one cohort by several researchers, including authors of this paper. In 2013, when we conducted Experiment 1, the group lived in a large outdoor aviary (12 × 9 × 5 m) with access to two adjacent roofed experimental compartments (each 3 × 4 × 5 m), where the experiment was conducted. In 2014, the group was moved to a larger outdoor aviary (15 × 15 × 5 m), which could be split in 3 compartments for the sake of Experiment 2 (see experimental procedure below). During the whole period the birds were fed a diverse diet containing meat, milk products, bread, vegetables, and fruits twice a day, and feeding protocols remained the same on testing days. In each aviary and testing compartment the birds had *ad libitum* access to water. All birds participated voluntarily in the experiments. Due to the non-invasive character of the experiments, the study complied with Austrian law and was authorized by the ethical board of the behavioral research group at the faculty of Life sciences, University of Vienna (case number: 2015-003). After the study the birds remained in captivity at the Haidlhof Research Station.

### Training

In the 3 months prior to Experiment 1 the subjects were trained to be isolated from the group in a testing compartment, and to exchange tokens (red plastic bottle tops) with an experimenter for rewards [pieces of frolic® (dog-food)]. Exchanging was shaped using positive reinforcement both with treats and verbal praise. After the birds had learned to directly exchange with an experimenter, they were trained to exchange through the fence of the test compartments and aviaries, and only at the site where a small red table was placed outside. Subsequently, the subjects were allowed to move freely between two test compartments, and were provided with 10 tokens in one compartment, while the red table was placed in front of the other compartment, and thus had to walk back and forth between the two compartments to exchange all tokens. When a subject managed to successfully exchange all 10 tokens within 10 min in 4 consecutive trials (in which the two test compartments were counterbalanced per individual), it proceeded to testing in Experiment 1.

Note that the ravens were never trained to transfer a token through the wire mesh in the door in between the two test compartments in Experiment 1, nor through the wire mesh between the compartments in Experiment 2. Thus, the animals learned only the procedure we used in both experiments, but not the response we measured.

### Experimental procedure and test conditions

#### Experiment 1

For Experiment 1 we selected fixed subject–partner dyads from the participating individuals from our group. We assumed that prosociality would be more prevalent among, or perhaps exclusive to, close social bonds. We therefore tried to select dyads with the best possible relationships, while also balancing dyads of kin and non-kin individuals, and dyads of same and different-sexed individuals. Note that at this age, the first social bonds form among ravens, but these are still strongly affected by kin relations (Braun and Bugnyar, 2012; Loretto et al., 2012). Subjects and their partners remained the same throughout the experiment, and to avoid reciprocity never changed roles. However, each subject did serve as a partner for a different individual other than his or her own partner (for actual subject-partner dyads see Table 1).

**Table 1:** **Name of subject and partner, sex and rank of subject, and the total number of transfers and subsequent exchanges by the partner (transfers/exchanges) in the 4 different conditions**.

**Subject**	**Partner**	**Sex**	**Rank**	**Total # of transfers/exchanges**			
				**Test**	**Social control**	**Non-social control**	Motivation control^*^
Laggie	Tom	m	1	0/-	0	5	0/40
Tom	Paul	m	2	0/-	0	0	0/40
Paul	Adele	m	6	10/9	1	5	0/40
Horst	George	m	4	0/-	2	2	0/38
George	Nobel	m	3	1/1	0	0	0/40
Louise	Horst	f	9	5/5	2	1	0/40
Nobel	Laggie	f	8	0/-	0	0	0/40
Adele	Louise	f	10	0/-	0	0	0/40

* *Exchanges performed by the subject itself*.

**Figure 1:**
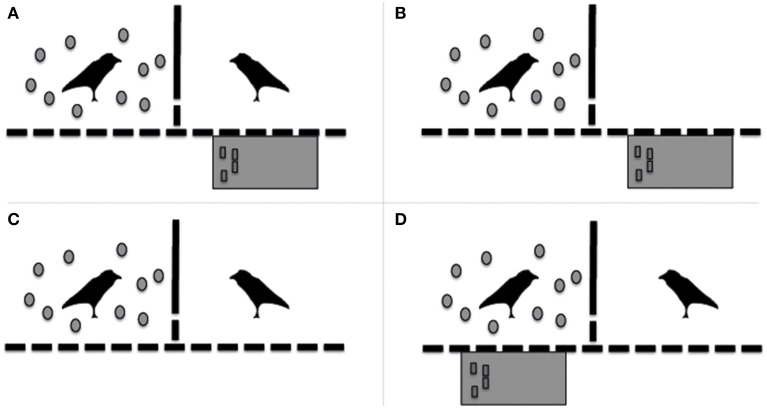
**Schematic representation of the different conditions in which the birds were tested in experiment 1. (A)** Test condition; **(B)** Non-Social Control condition; **(C)** Social Control condition; **(D)** Motivation Control condition.

Subjects (and their partners) were tested in two adjacent test compartments that were separated by wire mesh. Each bird was tested as subject 4 times in all 4 different conditions (test condition, social control condition, non-social control condition, and motivation control condition; Figure 1); i.e., 16 times in total. The order of conditions was semi-randomized such that each individual went through 4 subsequent runs of all four conditions, each run starting with a different condition. The subject and partner's position in a condition in either the right or left testing compartment was counterbalanced for each individual over the 4 repetitions. Neither subjects nor their partners were tested more than once per day.

##### Test condition (Figure 1A)

At the start of the test condition, the subject received 10 tokens (which were casted into the test compartment by the experimenter). However, the subject did not have the opportunity to exchange the tokens with the experimenter, since the red table (where they were trained to exchange) was placed in front of the adjacent test compartment where the partner was present. If the subject transferred a token to its partner through the wire mesh, the partner could subsequently exchange the token with the experimenter. If this chain of events occurred, only the (exchanging) partner would be rewarded with a piece (1/8th) of a dog biscuit (frolic®) and verbal praise. Consequently, the transfer of a token from the subject to the partner was considered a “prosocial” act.

##### Non-social control condition (Figure 1B)

To test whether prosocial acts were not due to an attempt to bring the tokens as close to the red table as possible, we performed a non-social control condition. This condition was similar to the test condition, however, without a partner present in the adjacent cage.

##### Social Control Condition (Figure 1C)

To test whether prosocial acts were not due to an attempt to object play, where two ravens play with the same object simultaneously (Heinrich, 1999), we performed a social control condition. This condition was similar to the test condition, however, the red table was not present at all, and both subject and partner had no opportunity to exchange tokens in this condition.

##### Motivation control condition (Figure 1D)

Since the subjects were not able to gain any rewards in either of the above-mentioned conditions, we also performed a motivation control condition. This condition was added to test- and both control conditions to both keep the subjects motivated and to test for general motivation. The motivation control condition was the same as the test condition, however, the red table was placed in front of the compartment of the subject instead of in front of the partner's compartment, thereby allowing the subject to exchange all 10 tokens it was provided with. Whenever, a subject exchanged a token it received a piece (1/8th) of a dog biscuit (frolic®) and verbal praise.

Each condition lasted until the 10 tokens were transferred or exchanged, or until a maximum of 10 min. The experiments were recorded using two Canon LEGRIA® HD-camcorders.

#### Experiment 2

**Figure 2:**
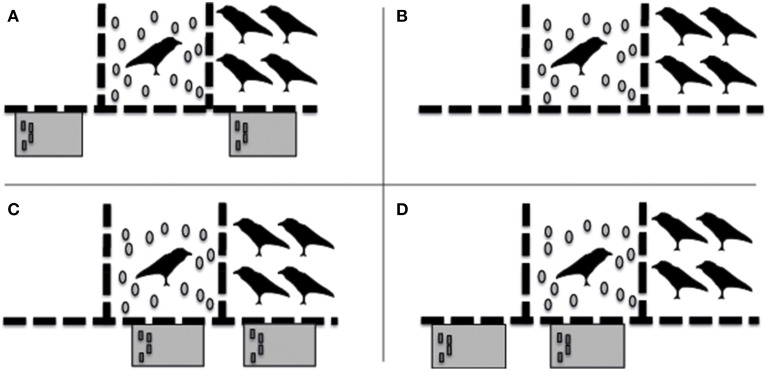
**Schematic representation of the different conditions in which the birds were tested in experiment 2: (A)** Test condition; **(B)** Social Control condition; **(C)** “Costly Prosociality” Test condition; **(D)** “Costly Prosociality” Control condition.

For Experiment 2 the group's home aviary was split into three compartments: two large compartments (each 6 × 15 × 5 m) on the outsides and one smaller compartment (3 × 5 × 5 m) in the middle, all separated by wire mesh. During testing the subject was always in the middle compartment, whereas the rest of the group (the remaining 9 birds) was either in the compartment to the left or to the right of the subject. Each bird was tested as subject 2 times in all 4 different conditions (non-costly “prosociality” test condition, social control condition, costly “prosociality” test condition, costly “prosociality” control condition; Figure 2); i.e., 8 times in total. The order of conditions was semi-randomized; i.e., 2 subsequent runs of all four conditions were carried out per individual; each run started with a different condition, and the different orders were pseudo-randomized across the 8 individuals. Also the position of the group, i.e., either to the left or to the right of the subject's compartment, was counterbalanced across the 8 individuals, and was counterbalanced per individual over the 2 repetitions. Subjects were tested only once per day.

##### Test condition (Figure 2A)

At the start of the test condition, the subject received 15 tokens (i.e., were casted into the test/middle compartment by the experimenter). However, the subject did not have the opportunity to exchange the tokens with the experimenter, since both red tables were placed in front of the adjacent compartments: one in which the group was present, one that was empty. If the subject transferred a token through the wire mesh to the compartment in which the group was present, a group member could subsequently exchange the token with the experimenter. If this chain of events occurred, only the (exchanging) individual would be rewarded with a piece (1/8th) of a dog biscuit (frolic®) and verbal praise. Consequently, the transfer of a token from subject to a group member was considered a “prosocial” act. Note that the red table in front of the empty compartment in this condition functions as a non-social control (cf. *non-social control condition* of Experiment 1).

##### Social control condition (Figure 2B)

To test whether prosocial acts were not due to an attempt to object play, like in Experiment 1, we performed a social control condition. This condition was similar to the test condition, although, no red tables were present, and both subject and its group members had no opportunity to exchange tokens in this condition.

##### Costly prosociality test condition (Figure 2C)

To test whether, if the ravens would be prosocial in the Test condition, they would also forego a reward for themselves to provide their group members with a reward, we performed a costly prosociality condition. In this condition, the subject could exchange all 15 tokens, or transfer them to the compartments in which its groupmembers were, where they could subsequently exchange the tokens themselves. The latter would be considered as a “costly prosocial act.”

##### Costly prosociality control condition (Figure 2D)

To test whether costly prosocial acts were not due to an attempt to object play, or due to an attempt to bring the tokens as close to the other red table as possible, we performed the costly prosociality control condition. In this condition, similar to the costly prosociality test condition, the subject could choose to exchange all 15 tokens itself, transfer it to an empty compartment that had a red table in front of it (cf. *non-social control condition* of Experiment 1), or to the compartment were the group was present, which however did not have a red table in front of it (cf. *social control condition* of Experiment 1).

As in Experiment 1, each condition lasted until all tokens were transferred or exchanged, or until a maximum of 10 min, and all experiments were recorded using two Canon LEGRIA® HD-camcorders. In Experiment 2 we needed two experimenters. Both experimenters took their position behind a red table (Test-, Costly Prosociality Test-, and Costly Prosociality Control- Condition) immediately after the tokens were casted into the subject compartment. They changed their position after 5 min, if the session lasted as long. Experimenters starting position; i.e., either behind the left or the right red table, was counterbalanced across the 8 individuals, and was counterbalanced per individual over the 2 repetitions. Both experimenters were also present during the Social Control condition at a distance of about 1.5 m.

### Behavioral data

Dominance rank data were deduced from monopolization experiments conducted during the corresponding time periods of both experiments (3 monopolization experiments per experimental period). In these monopolization experiments the study group was provided with two large pieces of partly frozen meat, which are highly valuable and easily monopolized. For half an hour per experiment we recorded all displacements and arranged these in a matrix with actors in rows and recipients in columns. Using MatMan (version 1.1; de Vries et al., 1993), we calculated Landau's linearity indices (h') and reordered the matrix to best fit a linear hierarchy (de Vries, 1995, 1998). We found a significantly linear hierarchy in both study periods (2013/Experiment 1: *h*' = 0.788, *n* = 10, *p* = 0.0024, based on 321 interactions and with 20% of unknown relationships, see Table 1; 2014/Experiment 2: *h*' = 0.709, *n* = 10, *p* = 0.0033, based on 528 interactions and with 16.56% of unknown relationships, see Table 2) with several rank changes between the two different periods.

**Table 2:** **Name, sex and rank of subject, and the total number of transfers to the group and subsequent exchanges by a group member (transfers group/exchanges) in the 4 different conditions**.

**Subject**	**Sex**	**Rank**	**Total # of transfers/exchanges**			
			**Test**	**Social control**	“Costly Prosociality” test^*^	“Costly Prosociality” control^*^
Laggie	m	1	0/-	0/0	0/19	0/30
Tom	m	2	0/-	0/0	0/30	0/26
Paul	m	3	2/1	0/0	0/30	0/15
Horst	m	5	0/-	0/0	0/30	0/30
George	m	4	0/-	1/0	0/30	0/15
Louise	f	7	0/-	0/0	0/30	0/30
Nobel	f	8	0/-	0/0	0/30	0/30
Adele	f	10	0/-	0/0	0/30	0/29

* *Exchanges performed by the subject itself*.

Since ravens readily cache/hide food and non-food items (Bugnyar and Kotrschal, 2002) we coded if and how many tokens each individual cached per condition.

Finally, the behavior of the partners (Experiment 1) and the rest of the group (Experiment 2) in the tests was coded from the videotapes and categorized as (a) interest (i.e., the time an individual spent in front of the wire mesh separation), and (b) attention-getting behaviors (i.e., all vocalizations and pecking at any object that creates a sound).

### Analysis

Choices were coded live by JJMM and recoded from the videos by ML. Inter-rater reliability was perfect (Cohen's kappa = 1).

To assess whether the ravens in our study were prosocial, we compared the number of tokens they transferred from their own compartment to that of their partner(s) in the test and control conditions using Friedman tests, and performing *post-hoc* Wilcoxon signed ranks tests. Similarly, partner behavior was compared between conditions using Wilcoxon signed ranks tests. Comparisons on an individual level were calculated using Chi squared tests with Yates correction. All analyses were performed in IBM SPSS Statistics v.20 for Mac OS, with α set to 0.05. All reported *p*-values are 2-tailed.

## Results

### Experiment 1

All ravens were very motivated to participate in Experiment 1. In the motivation control sessions each raven exchanged (almost) all tokens (Mean total = 39.75 ± S.E. 0.25 out of 40; Table 1), and in general exchanged the 10 tokens per session in less than 2 min (Mean time necessary to exchange the 10 tokens per session = 109.3 s ± S.E. 15 s).

**Figure 3:**
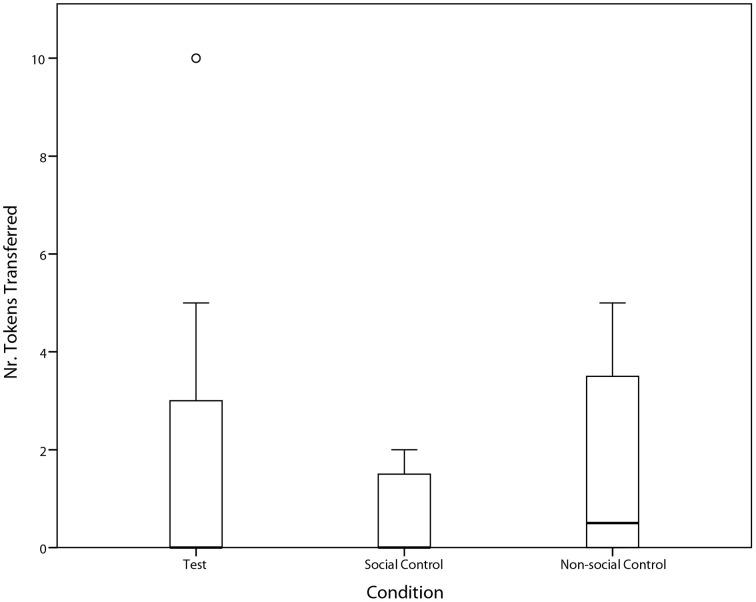
**Median ± Quartiles and 95% confidence intervals of the number of tokens transferred from subject to partner in Test-, Social Control, and Non-Social Control Condition**.

Overall, the ravens were not prosocial; i.e., we did not find a significant difference in the amount of token transfers between the test condition and the two control conditions (Friedman test: χ^2^ = 1.06, *n* = 8, *p* = 0.589; Figure 3). Nevertheless, three individuals did spontaneously transfer tokens in the test condition (see Table 1), and one did so significantly more so than in the control conditions (Paul: Yates χ^2^ = 4.06, *df* = 1, *p* = 0.049; Table 1 and Supplementary Video 1). Specifically, Paul performed significantly more token transfers in the test condition than in the social control condition (Yates χ^2^ = 6.75, *df* = 1, *p* = 0.009), but not compared to the non-social control condition (Yates χ^2^ = 1.31, *df* = 1, *p* = 0.25).

Rather than transferring tokens to a partner, if the birds were not able to exchange their tokens themselves, they cached about half of the tokens they were provided with in the sand (Mean ± S.E. = 18.63 ± 1.19 out of 40), irrespective of the condition they were in (Friedman test: χ^2^: 2.00, *n* = 8, *p* = 0.368).

Regarding partner behavior, we found no evidence for partners paying more attention to the subjects in the test condition than in the social control condition; if anything, there was a non-significant trend that they paid less attention (Wilcoxon signed ranks test: *T^+^* = 31.0, *n* = 8, *p* = 0.069), most likely because they were distracted by the experimenter sitting in front of them during the test condition. Similarly, partners did not show a significant difference in the amount of attention-getting behaviors between the test and social control condition (Wilcoxon signed ranks test: *T^+^* = 26.0, *n* = 8, *p* = 0.26).

### Experiment 2

As in Experiment 1, all ravens were very motivated to participate in Experiment 2. Whenever they had the opportunity to exchange tokens themselves (i.e., in conditions C and D, see Figure 2), they exchanged almost all of them with the experimenter (Mean total = 27.13 ± S.E. 1.38 out of 30; Table 2) and in general exchanged the 15 tokens per session in about 4 min (Mean time necessary to exchange the 15 tokens per session = 242 s ± S.E. 32.5 s). None of the birds transferred a token to any of their group members in these “costly prosociality” conditions, and only 1 bird did so in the “normal” prosocial test condition (2 tokens), whereas 1 bird transferred one token in the social control condition (Table 2). Consequently, there was no significant difference in the number of tokens transferred between the different conditions (Friedman test: χ^2^ = 2.00, *n* = 8, *p* = 0.572). Interestingly, however, the one bird that did transfer tokens in a test condition in Experiment 2 (Paul), was the same bird that did transfer most tokens in the test condition of Experiment 1.

Similar to Experiment 1, whenever the birds could not exchange the tokens themselves, they cached about half of all tokens in the sand (Mean ± S.E. = 13.75 ± 1.98 out of 30), irrespective of the condition they were in (Friedman test: χ^2^: 2.00, *n* = 8, *p* = 0.368).

Regarding partner behavior, we found no evidence for the group paying more attention to the subjects in the test conditions than in control conditions; i.e., there was no difference in the amount of birds that spent time in front of the wire mesh between the test compartments (Wilcoxon signed ranks test: *T^+^* = 4.5, *n* = 8, *p* = 0.206), nor in the amount of time they spent nearby (Wilcoxon signed ranks test: *T^+^* = 10, *n* = 8, *p* = 0.263). Similarly, partners did not show a significant difference in the amount of attention-getting behaviors between the test and social control conditions (Wilcoxon signed ranks test: *T^+^* = 21.0, *n* = 8, *p* = 0.674).

## Discussion

Our results show that in our experimental set-ups subadult ravens do not regularly help their conspecifics to gain access to food. Given these results, in accordance with Di Lascio et al. (2013), we cannot conclude that ravens are prosocial, at least during the first years of their life. This is in apparent contrast to what has been reported for several primate species (reviewed in Cronin, 2012; but see also Amici et al., 2014) and for closely related jackdaws (Schwab et al., 2012; for a review on all these species see Cronin, 2012).

The discrepancy between the two corvid species may be due to different socio-ecological evolutionary pressures like for example the different breeding systems of jackdaws and ravens. Whereas, raven pairs breed in large territories (Heinrich, 1999), jackdaw pairs breed in colonies, and their subsequent higher intraspecific tolerance and dependence on each other regarding nest defense against predators (Johnsson, 1994), may have led to the evolution of prosociality in the jackdaws only (cf. Burkart et al., 2014). However, non-breeding ravens, like the ones we tested here, are known to congregate in large, highly social flocks, show high intraspecific tolerance, and even form strong and stable social bonds (not only with future pair-mates) (Fraser and Bugnyar, 2010). Comparative work on various corvid species with varying degrees of allo-parental care should, however, elucidate whether the observed pattern is a general one that corroborates the cooperative breeding/alloparental care hypothesis (Burkart et al., 2007, 2014). In addition, the discrepancy between the two corvid species may also be due to other ecological differences between the species. For example, jackdaws do not cache food, whereas ravens regularly do (de Kort and Clayton, 2006), and in this paradigm also had the opportunity to do so with the valuable tokens.

Moreover, there may be several other reasons why the ravens in these experiments did not take the opportunity to help their conspecifics to gain access to food. First, the ravens in these experiments might not have understood the paradigms sufficiently. While the birds were adept at moving between the two compartments to exchange tokens themselves, they had no prior experience exchanging tokens with or transferring tokens to conspecifics. Alternatively, in the test conditions the ravens may have expected to be able to exchange tokens themselves at a later point in time, which would explain their caching behavior in those conditions, or they could not inhibit the urge to cache in these conditions. Insufficient understanding of the task at hand has also been suggested to explain why chimpanzees did not help others, while human children did so in a similar helping paradigm (Warneken and Tomasello, 2006). In support of this explanation, we did observe, albeit sporadically, that during the test conditions and during the different control conditions some of our birds did push tokens through the wire mesh on the side of the experimenter, as to exchange the token with the experimenter, although neither the appropriate red table nor the experimenter were present there (see Figures 1, 2). Additionally, partners also did not show more interest in the subjects, nor tried to get their attention more in conditions where they could exchange a token than in conditions in which they did not have such opportunity. Alternatively, this hypothesis would predict that if given some experience with the characteristics and possible outcomes of the set-up, the ravens might have become more prosocial. However, although two of the three ravens that did transfer some tokens to their partners in two consecutive test-sessions of Experiment 1, they ceased to do so in the following test-session. Nevertheless, it was one of these ravens that also transferred tokens to the group in the test-condition of Experiment 2, albeit at a non-significant level, which might support the hypothesis and suggests that this individual was the only one that had some understanding of the task at hand.

Second, the unequal reward distribution between partner and subject (i.e., only the partner would receive a reward), might have inhibited subjects to be prosocial already at the start of the study, and might have caused individuals that did try to exchange tokens to cease this behavior when they discovered there is nothing to gain for themselves. Ravens are sensitive to disadvantageous inequity (Wascher and Bugnyar, 2013), and this inequity aversion may have competed with prosocial preferences (Brosnan et al., 2010; Cronin, 2012). For example, in capuchin monkeys it has been shown that a disadvantageous reward distribution for the actor in comparison to its partner (0/1) does reduce prosocial preferences in comparison to a set-up in which the actor and the partner are rewarded equally (1/1) for a prosocial choice of the actor (Brosnan et al., 2010). Similarly, long-tailed macaques were shown to be prosocial toward kin at no cost to themselves (Massen et al., 2010), yet did not choose to be prosocial toward their kin when this choice forces them to forgo a high-quality reward (Sterck et al., 2015). Therefore, it would be very interesting to see how ravens would behave in a 1/1 prosocial choice task.

Although the ravens in our experiment were not prosocial in general, acts of prosocial helping did spontaneously occur, albeit very little and not always significantly more than in the control conditions. The few isolated cases we report here do not allow us to analyze effects of the identity of the tested individual (cf. Massen et al., 2010) and the composition of the dyad (cf. Massen et al., 2011) on prosociality. Especially since only one individual showed consistent helping in both the dyadic and group experiment, though not more than the control conditions in the group experiments and only significantly more than one (out of two) control conditions in the dyadic experiment, hypotheses about underlying motivations are highly speculative. As mentioned above, it could be that only this individual understood the task at hand. Alternatively, since he was paired with a female, it might be that this individual was trying to form a pair bond with the female through behaving prosocially toward her. This would correspond to his developmental stage (Braun and Bugnyar, 2012) and also to the results found in jackdaws, where more cooperative acts were reported for mixed-sex pairs than for same-sex pairs (Schwab et al., 2012). However, this was not the only mixed-sex pair we tested, further, this subject also transferred tokens, albeit only 2, to other males in the group test of Experiment 2. Nevertheless, it may be particularly interesting to test also adult ravens in our set-up, as the interdependence of the monogamous pair-bond (Braun and Bugnyar, 2012) may facilitate prosocial tendencies among the birds. Finally, it is interesting to note that the one bird that consistently transferred tokens to conspecifics was relatively weakly integrated in the group (*unpublished data*). It may be that through behaving prosocially he was trying to become better integrated, much like primates groom up the hierarchy for tolerance (Seyfarth, 1977; von Bayern et al., 2007).

In our set-up, we chose to avoid the possibility to immediately reciprocate, in order to test true prosocial preferences; i.e., without the possibility of an immediate return of the investment. However, it remains interesting what would have happened if we had allowed the ravens to reciprocate. For example, in basically the same set-up, though with the ability to reciprocate, Pelé et al. (2009) showed that in contrast to our ravens, bonobos, and orang-utans did frequently transfer tokens, whereas, similar to our ravens, chimpanzees and gorillas rarely did (Pelé et al., 2009). Moreover, the transfers of two particular orangutans seemed to be calculated (Dufour et al., 2009; but see Pelé et al., 2009). Similarly, Norway rats rely on obtained benefits to adjust the level of help they return reciprocally (Dolivo and Taborsky, 2015), although it has also recently been shown that Norway rats can show spontaneous prosociality (Hernandez-Lallement et al., 2015). In addition, modeling studies (e.g., Gintis et al., 2003; Forber and Smead, 2014) suggest that in combination with a sense of fairness, a trait recently reported to exist in ravens (Wascher and Bugnyar, 2013), through reciprocity even the sporadic acts of helping reported in this study might lead to cooperation on the scale ravens display in their natural lives (e.g., Fraser and Bugnyar, 2012; but see Cronin et al., 2010). The evolution of spite, in turn, even enhances the evolution of cooperation through fairness (Gintis et al., 2003; Jensen, 2010; Forber and Smead, 2014). So far, however, there is no experimental data supporting the notion of functional spite in non-human animals, suggesting a distinction between humans and non-human animals that may explain human “hyper-cooperativeness” (Jensen, 2010).

In contrast to other studies (e.g., Horner et al., 2011; Schwab et al., 2012) that showed that the behavior of the partner could predict whether an actor was prosocial or not, our data do not support such a notion. Similarly, we did not find any effect of the identity of the subject or of the characteristics of the tested dyad, as was shown in long-tailed macaques, for example (Massen et al., 2010, 2011). However, given the scarcity of prosocial acts in our results, and consequently little variation in prosociality among our birds, we feel that we cannot draw any conclusions from abovementioned null-results.

In sum, we showed that in a helping set-up sub-adult ravens do not consistently exhibit prosocial preferences. Future research should illuminate whether this absence reflects an evolutionary deviation from the closely related jackdaws, and from the more distant primate species in which prosociality has been revealed experimentally, or whether the difficulty of the task at hand and the unequal reward distribution for the actor may have inhibited existing prosocial preferences among the ravens. Nevertheless, we showed that prosocial helping can occur spontaneously in ravens, and we suggest that even these sporadic instances, in combination with a sense of fairness, might lead to stable cooperation in this species.

### Conflict of interest statement

The authors declare that the research was conducted in the absence of any commercial or financial relationships that could be construed as a potential conflict of interest.
